# Gender Neutral Language in (Greater) Buenos Aires, (Greater) La Plata, and Córdoba: An Analysis of Social Context Information Using Textual and Temporal Features

**DOI:** 10.3389/fsoc.2022.805716

**Published:** 2022-03-17

**Authors:** Olga Kellert

**Affiliations:** Romance Department, University of Göttingen, Göttingen, Germany

**Keywords:** Twitter, textual analysis, speech act analysis, gender neutral language, Greater Buenos Aires, Córdoba

## Abstract

In this article, I explore Twitter data to analyze Gender Neutral Language (GNL) in (Greater) Buenos Aires, (Greater) La Plata, and Córdoba. The goal is to characterize the social context behind GNL. Social context analysis of social media data is challenging given that this data type does not contain the social characteristics of its users and the circumstances under which the tweets were written. In order to fill this gap, I will derive the social context information from textual and temporal features by analyzing the names of locations, companies, and people used in the text and relating these entities to the message of the tweet. The analysis of temporal features will give us insights into the correlation between language use and social events. Our results show that the general characterization of the social context behind GNL is associated with socio-economically rich areas in city centers. Users of GNL in the investigated areas address certain groups of people with words that express familiarity and close social relationships, such as those meaning “friends” and “neighbors” and that give them information about a political, cultural, or social event or concerning commercial products/services. The temporal analysis by month supports this characterization by showing that certain political and social events induce a higher frequency of GNL. This paper contributes to previous research on GNL in Argentina by testing existing hypotheses quantitatively. The new discovery presented here is that political activism is not the only language context in which GNL is used in social media and that GNL is not exclusively used in big cities of Argentina but also in smaller cities.

## Introduction

Over the past years, the use of GNL, that is, linguistic expressions that include all sexual groups, also known as inclusive gender language, has increased (see Tosi, 2021). This phenomenon has been studied extensively from different perspectives, such as from the sociolinguistic, political, cultural, educational (see Mozdzierz, 1999; Pauwels, 2003; Motschenbacher, 2010; Prewitt-Freilino et al., 2012; Hord, 2016; Melamud, 2017; Morales, 2018; Slemp, 2020; Zimman, 2020; Erdocia, 2021; Kirey-Sitnikova, 2021; Tosi, 2021), and, more recently, also from the computational point of view (Orǎsan and Evans, 2007; Heng and Dekang, 2009; Nguyen et al., 2016).

The present study investigates GNL in the Spanish-speaking country of Argentina. Spanish is a Romance language with grammatical gender; that is, nouns and elements that agree with nouns, such as determiners, adjectives, and pronouns express gender linguistically with a feminine or masculine marker (Slemp, 2020). The gender of animate nouns, such as those denoting humans, corresponds to the apparent biological sex of the referent (Slemp, 2020), with two alternate forms, male and female (e.g., *el chico* “the boy” and *la chica* “the girl”). The most frequent masculine ending of nouns referring to male humans is *-o*, and the most frequent feminine ending referring to female humans is *-a*. Some nouns referring to humans mark gender only by agreeing elements such as determiners (e.g., *el/la artista* “the artist”) (Slemp, 2020). In writing, there are a number of ways to represent such masculine/feminine pairs in a gender-neutral way in Spanish, such as writing one gender ending after another as in e.g., *amigo/a* “friend” or as in *amiga(o)* “friend” or by using single forms that are gender-neutral, namely *-@, -e, - x*. The latter marker, *-x*, was first introduced by Latin American immigrants in the United States (Morales, 2018; Slemp, 2020). *Latinx* is an “explicit incorporation” of gender minorities (Vidal-Ortíz and Martínez, 2018, p. 394, Slemp, 2020). Students in colleges and universities in the United States use this ending quite frequently. Contreras (2017) argues that the term *latinx* should be reserved for members of the LGBTQ+ community (Slemp, 2020). As the suffix *-x* is problematic for pronunciation, a new marker for gender inclusive language, *-e*, has appeared to avoid overt gender markings such as *-o* and *-a*, such as *latine* instead of *latino* and *latina* (Slemp, 2020). The popularity of the *-e* marker in comparison to *-@* and *-x* is due to its easy pronunciation according to Galperín, a professor of literature at the Torcuato di Tella university in Buenos Aires, as reported by *The New York Times* (April 15, 2020).

It is generally assumed that GNL is defined as “a way of speaking that does not perpetuate gender-based stereotypes, and includes all gender identities” (Nissen, 2013; Vidal-Ortíz and Martínez, 2018; Slemp, 2020, p. 1, among others). Both the European Commission and UNESCO have released guidelines with suggestions on how to use GNL in many European languages (UNESCO, 1999; Slemp, 2020). Their guidelines are not mandatory. Not all European countries have followed these guidelines for the institutionalization of GNL. In France, for instance, the use of GNL is not institutionalized. In Spain, GNL was institutionalized in 2018 (Erdocia, 2021). In many of the documents describing the goals of institutionalization of GNL in Spain, the use of non-neutral gender such as masculine generics, are said to foster inequality against women (Erdocia, 2021). The use of GNL is seen as contributing to greater equality between genders. However, there is still a debate as to the recognition of GNL in Spain socially, politically, and linguistically (Erdocia, 2021). According to a YouTube corpus compiled by Slemp et al. (2019), Spanish-speaking countries in which GNL appears the most are Spain and Argentina (Slemp, 2020). A similar result has been shown in a study on Twitter, a social media platform on which users post short messages known as “tweets,” conducted by *la Fundación del Español Urgente* (“Foundation of Urgent Spanish”) and *el Instituto de Ingenier*í*a del Conocimiento* (Institute of Knowledge Engineering) from April 2020 (Fundéu and Instituto de Ingeniería del Conocimiento, 2020). In this study, four words—*niños, nosotros, todos y ciudadanos* “kids, we, all and citizens”—are investigated in 21 Spanish-speaking countries over a period of 20 days. The overall results show that Spain and Argentina are among other five top countries using GNL. The results also show that 1.19% of all tweets use GNL (which breaks down as *-@* 50.58%, *-x* 31.44%, and *-e* 17.98%). The form *-x* is used more in Argentina and Costa Rica, whereas the form *-@* is used more in Chile, Spain, and Venezuela. The form *-e* occurs more often in Argentina, Puerto Rico, and Uruguay.

Argentina, the focus of the present study, is particularly interesting in this context due to recent social and political events connected to GNL. Argentina stands out as to how “widely embraced the new forms [GNL] have been not just among activists but also in academic and government spheres” (Politi, 2020) (*The New York Times*, April 15, 2020). Various political and social events may have triggered the widespread discussion in society and an increase of GNL in Argentina. In 2018, a young journalist used gender-neutral expressions in a television interview, which earned her criticism (Schmidt, 2019) (*The Washington Post*, December 5, 2019). This event triggered widespread discussion in society (Slemp, 2020). In 2019, the use of gender-neutral expressions started to gain more official acceptance (Slemp, 2020). *The New York Times* reports that GNL has started to be used in some governmental departments in Argentina after Alberto Fernández became president in 2019, who supported the use of GNL in official documents (*The New York Times*, April 15, 2020). However, no directive to use of GNL (such as *-e*) in official documents was achieved in Argentina (Slemp, 2020). According to Victoria Donda, the head of the National Institute Against Discrimination, Xenophobia and Racism in Argentina, the use of inclusive marker *-e* could lead to confusion in poorer neighborhoods, where people are not used to hearing this marker, according to the same *New York Times* article. According to Moure (2018), GNL in Argentina is “consciously put forth and planned by a minority group, usually characterized as educated, middle class and urban” (Tosi, 2021; citing Moure, 2018). GNL is associated with urban environments, realms of activism, and certain public administration sectors in Argentina (see Moure, 2018).

The present study aims at investigating how the characterization just presented of GNL usage in Argentina is reflected in the data, that is, at exploring whether we find evidence in the data that the hypothesis from the literature that I summarize here below is correct:

GNL in Argentina is mainly used by a specific group of people characterized as urban, middle class, and educated who are associated with political activism or serve in the public administration.

In order to probe this hypothesis, I will use social media data from Twitter. Twitter provides a large amount of data with valuable metadata such as temporal information. This information can provide us with answers to questions when gender expressions start to diffuse. Big temporal changes in GNL use can be connected to social and political events such as the electoral campaign of Alberto Fernandez and the time after his campaign and can thus provide more contextual information to the given phenomenon. In this article, we will thus study the relation between temporal variable and content analysis.

In spite of these advantages, Twitter data may contain certain biases, which are already present by the fact that not all people are using the platform. To evaluate the degree of biases on Twitter with respect to gender distribution and diffusion, I will use a comparative method by comparing the results on gender distribution in a number of large and smaller cities. Finally, I will compare our results with previous results in research on GNL in Argentina. Another challenge for research on social context information in GNL is that social media do not provide complete information about their users such as age and socioeconomic status, also known as social variables (Labov, 2006). I will fill this gap using textual and temporal analysis that will provide us answers concerning the social circumstances under which GNL is used.

The paper is structured as follows. Section 2 presents the Data and Methods. Section 3 presents the Results from the textual analysis. Section 4 shows temporal changes in gender-neutral usage. And finally, Section 5 compares the results with the state of the art and provides an outlook for future research.

## Data and Methods

This study examines data from the two largest cities in Argentina—Buenos Aires (BA) and Córdoba—as well as the surrounding area of the city of Buenos Aires (GBA) and of the city La Plata (GLP). GBA is an urban agglomeration comprising the city of Buenos Aires and 24 surrounding districts that belong to the Province of Buenos Aires. According to the National Institute of Statistics and Census of Argentina from 2010 the 10 biggest most populated cities of GBA are: BA, Merlo, Quilmes, Banfield, José Clemente Paz, Lanús, Gregorio de Laferrère, Hurlingham, Berazategui, González Catán. BA has 2,776,138 inhabitants and the number of inhabitants in other cities ranges between 244,168 and 89,073 (National Institute of Statistics Censuses, 2003). The city La Plata has 643,133 inhabitants. Córdoba has 1,391,298 inhabitants.

The tweets were further filtered to include only those written in Spanish (i.e., Lang = “es”), and the time-span of the available data included late 2017 up to early 2021, precisely from November 2017 through March 2021. A small proportion of tweets also contain geographic coordinates using the Global Positioning System (GPS). These tweets correspond to somewhat <1% of the total number of tweets. Twitter announced it would eliminate the geolocation information starting in mid-2019 (Kruspe et al., 2021). However, some tweets still contained the geolocation information in the meta-data accessible through the Twitter API (Kruspe et al., 2021).

For this study, I only used geolocated tweets for the purposes of a follow-up study aiming to compare social context information derived by content analysis presented in this article with social context information derived by geolocation information (Kellert and Matlis, under review^1^). I filtered out all geolocated tweets that correspond to GBA and GLP, which correspond to a longitude range of −58.531725 to −58.355148, and a latitude range of −34.538162 to −34.705446.

The time period gave us 154,599 geolocated tweets in Córdoba, 1,065,614 in the city of Buenos Aires, and an additional 1,930,987 in Greater Buenos Aires (GBA) and Greater La Plata (GLP) (henceforth GBALP). I further filtered out tweets associated with fewer than five digits in the number representing precise geolocation information, in order to be sure that the geolocation was precise (Kellert and Matlis, under review^1^).

I defined contrast pairs of gender-neutral and non-gender-neutral expressions, calling the latter expressions just (overt) gendered expressions. Gender-neutral expressions were defined by characters such as *-@ or -x*+*delimiting space* to catch singular expressions and by *-@s* or *-xs*+*delimiting space* in order to catch plural expressions, such as *Querid@s amig@s* “Dear friends_[neut]_.” Overt gendered expressions were defined by –*a* or *-o* + *delimiting space* or by –*as* or *-os*+*delimiting space*, as in *Queridas amigas* “Dear friends_[femininegender]_” or *Queridos amigos* “Dear friends_[masculinegender]_.” I also included two frequent tokens with a gender-neutral plural -*es* such as *amigues* and *chiques* “friends, girls/boys_[plural neut]_.”

gender-neutral = [“rxs”, “r@s”, “todx”, “tod@”, “unx”, “un@”, “nxs”, “n@s”, “lxs”, “l@s”, “in@”, “inx”, “much@”, “muchx”, “amigues”, “amig@”, “amigx”, “l@”, “lx”, “s/-a”, “s/a”, “r/-a”, “r/a”, “chiques”, “amigues”, “lxs”, “l@s”].

gendered = [“ina”, “ino”, “inas”, “inos”, “los”, “las”, “el”, “la”, “chicos”, “amigos”, “chicas”, “amigas”, “otro”, “otra”, “mucha”, “mucho”, “un”, “una”, “unos”, “unas”].

It is important to note that the tokens representing the set of (overt) gendered expressions do not refer only to humans with an explicit masculine or feminine gender, e.g., *mucho*_[masculinesingular]_ *trabajo*_[mascunlinesingular]_ “a lot of work.” As our focus lies on gender-neutral expressions and not on gendered expressions, the disambiguation of gendered expressions is not relevant in our study.

After the definition of the token sets representing different types of gender, we automatically extracted tweets on the basis of linguistic features as defined above from the content of the text of geotagged tweets.

In order to interpret and analyze the content of the tweets, I focused on different linguistic aspects of the tweets. There are different types of linguistic analyses that focus on different aspects of the meaning of linguistic expressions such as the information structure, which deals with focus and background information (e.g., it's Mary that I love and not Peter.) or topic and comment structures (e.g., as to Mary, I love her), discourse analysis and argument structure that investigate the relationship of many sentences that form a pragmatic unit, speech act analysis and lexical semantic analysis (Lyons, 1995, for an overview). As the main goal of this study is to probe the hypothesis from the literature defined in Section 1, namely that GNL is mostly used in the realm of political activism (Moure, 2018), i.e., twitters have the intention to inform or to invite to events associated with political activism, a speech act analysis seems to me the most important aspect of the content analysis. The main semantic aspect will be to focus on twitters, their intentions and their addressees. The utterance *Have a good day, Mary!* can be interpreted as an act of wishing a good day to the addressee with the name Mary. Typical written markers of speech acts are punctuation marks such as question or exclamation marks at the end of the sentence (Grice, 1989). Other linguistic features are speech act verbs (e.g., to inform, ask, offer, etc.) or syntactic features that correspond to particular speech acts, such as subject–verb inversion in questions (Grice, 1989), declarative markers, modal adverbs and modal verbs, etc. (Grice, 1989). Speech act analysis is a functional analysis of linguistic expressions as it connects linguistic markers and expressions to the pragmatic function and the utterance context (see also Halliday and Matthiessen, 2014 for a functional analysis).

In order to analyze the content of the tweet messages, I especially payed attention to the lexical words used in the tweets and whether they belong to the political domain or political activism, i.e., words such as *demonstration, rights, protest, political movement, vote*, etc.. Moreover, I particularly focused on event nouns and event verbs (e.g., *a protest* or *to meet*) in order to find out whether all or many of them are associated with political events or political activities. Names of locations of companies/institutions and people were then analyzed to see whether they fit the hypothesis from the literature that political activism is the most important factor driving the use of GNL.

For the textual and lexical analysis, I filtered out the most frequent tokens and collocations. Frequency lists have just a filtering function for most frequent words for my lexical semantic and textual analysis. The automatic method thus does not replace the manual textual analysis. It just serves to focus on the most frequent words. I used one of the most common tools for frequency list generation from NLTK, a common library in Python (see Bird et al., 2009). I filtered out most frequent single tokens and pairs of tokens (collocations). Tokens are strings separated by some delimiter such as space. Collocations allow us to gain more specific information about tokens by examining their frequent neighbors (e.g., sports articles is more specified than just articles).

However, the NLTK tokenization algorithm considers @ inside words as a separate token; so, for example, *para tod@s l@s artistas* “for all the artists_[neut]_” creates a set of tokens: [“para,” “tod,” “@,” “s,” “l,” “@,” “s,” “artistas”]. Tokenization using NLTK thus creates ambiguity with @ used for other functions, such as reference to names on Twitter. In order to avoid this ambiguity, I filtered out the gender-neutral function of @ for our study.

Our final step was a temporal analysis of tweets in order to see whether the gender-neutral usage correlates with certain temporal features that might be explained by social and/or political events.

## Results

This section presents the findings of the study.

### Frequency of ±Gender-Neutral Expressions

The frequency of gender-neutral expressions in Table 1 is expected to be lower than that of gendered expressions in all areas investigated. This is due to many factors. First, the use of gender-neutral expressions is relatively new, and second, gendered expressions do not all refer to sexual gender as mentioned in Section Data and Methods. The lower percentage of gender-neutral use in Córdoba in contrast to other areas reflects the lower usage of tweets with gender in general in this area. It is surprising though that there are nine times less tweets with overt gendered expressions in Córdoba than in Buenos Aires, although Córdoba is only two times smaller in terms of inhabitants number than Buenos Aires. More research is needed to understand this bias.

**Table 1:** Frequency of tokens representing ± neut. gender in all three corpora.

**Gender**	**GBALP**	**Buenos Aires**	**Córdoba**
+Neut	2,852 (0.45%)	1,542 (0.43%)	144 (0.37%)
Gendered	627,158 (99.55%)	356,001 (99.57%)	38,477 (99.63%)
Total	630,010 (100%)	356,543 (100%)	38,621 (100%)

### Textual Interpretation

In this section, I will look at the social context of GNL usage derived by textual analysis.

Table 2 shows the 100 most frequent collocations in all areas investigated, while Table 3 shows the 100 most frequent single tokens. I have highlighted all tokens containing gender-neutral expressions with __ and all tokens referring to locations, companies/shops/institutions, and people in bold. The English translation is represented by ‘' (see Supplementary Material).

**Table 2:** Most frequent collocations.

**Buenos Aires**	**Córdoba**
[(“@,” “s”), **(“Buenos,” “Aires”)**, (“tod,” “@”), **(“Ciudad,” “Autónoma”)**, (“amig,” “@”), **(“Autónoma,” “Buenos”)**, (“l,” “@”), (“todxs,” “lxs”), (“Gracias,” “todxs”), (“Feliz,” “día”), **(“Plaza,” “Mayo”)**, (**“Distrito,” “Federal”)**, (“lxs,” “trabajadorxs”), (“Nos,” “vemos”), (“lxs,” “compañerxs”), **(“Bs,” “As”), (“Capital,” “Federal”)**, (“Muchas,” “gracias”), **(“San,” “Telmo”), (“Puerto,” “Madero”)**, (“fin,” “semana”), **(“R**í**o,” “Colorado”)**, (“s,” “l”), **(“LNP,” “San”)**, (“lxs,” “pibxs”), (“ell,” “@”), **(“Teatro,” “R**í**o”), (“Art,” “Tattoo”), (“Ganesha,” “Art”)**, (“MODA,” “ 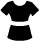 ”), (“SIGAS,” “automáticamente”), **(“Tattoo,” “Studio”)**, (“automáticamente,” “participan…”), (“unipersonal,” “payase”), (“ 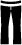 ,” “ 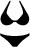 ”), (“ 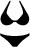 ,” “ 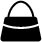 ”), (“ 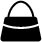 ,” “ 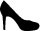 ”), (“ 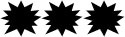 ,” “ 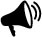 Se”), **(“Colorado,” “teatro/escuela”), (“Junta,” “Interna”)**, (“ME,” “GUSTA”), (“además,” “PRE-VEN…”), (“pongas,” “ME”), (“sigo,” “contando”), (“▾▾▾▾▾▾▾,” “SEGUINOS”), (“curso,” “¡EMPRENDEDORES”), (“on,” “line”), (“saludar,” “agradecer”), (“ 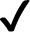 ,” “Soy”) (“ 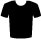 ,” “ 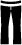 ”), (“ 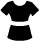 ,” “ 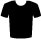 ”), (“lxs,” “chicxs”), **(“Telmo,” “Ciudad”)**, (“invitamos,” “pongas”), **(“Desde,” “Junta”)**, (“GUSTA,” “NOS”), (“NOS,” “SIGAS”), (“Quiero,” “saludar”), (“ 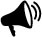 Se,” “acerca”), underline (“nosotr,” “@”), (“L,” “@”), (“Federal,” “Argentina”), (“69,” “paz”), (“vino,” “nomas”), **(“Facultad,” “Derecho”)**, (“Buen,” “día”), (“#,” “@”), (“primera,” “vez”), (“trabajadorxs,” “INCAA”), **(“Derecho,” “UBA”)**, (“Me,” “sigo”), **(“Caballito,” “Buenos”), (“Club,” “69”)**, (“Acaba,” “publicar”), **(“Botica,” “Ángel”)**, (“Maquillador/a,” “Profesional”), **(“Medrano,” “1647”), (“NIKE,” “ZOOM”), (“Norita,” “Cortiñas”)**, (“Partido,” “Piquetero”), **(“TANGO,” “BUTOH”)**, (“Trayecto,” “Maquillador/a”), (“ZOOM,” “HYPERATACK”), (“diría,” “ 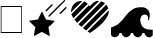 ”), (“edades,” “niveles”), (“elisabetsauro,” **“pinallieugenioangel”)**, (“homenajeando,” “abanderada”), (“importar,” “edades”), (“profesionales!!,” “Encontrálas”), (“psicomotricidad,” “fina”), (“Ángel,” “ 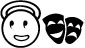 ”), (“compañer,” “@”), (““,” “Quiero”), (“buena,” “onda”), **(“Palermo,” “Soho”)**, (“Muy,” “feliz”), (“Etiqueta,” “amig”), (“Encontrálas,” “tienda”)]	[(“@,” “s”), (“tod,” “@”), **(“Louise,” “L”)**, (“dejó,” “querida”), (“querida,” **“Louise”)**, (“tratamientos,” “dejó”), (“Buenos,” “días”), (“serie,” “tratamientos”), (“l,” “@”), (“ONLINE,” “atender”), (“Plan,” **“X5”**), (**“X5,”** “prioridad”), (“afirmaciones,” “positivas”), (“atender,” “todas…”), (“manera,” “ONLINE”), (“positivas,” “mujeres”), (“prioridad,” “salud”), (“trabajando,” “manera”), (“L,” “Hay”), (“LO,” “MEREZCO”), (“ME,” “LO”), (“Seguimos,” “serie”), (“En,” “Plan”), (“seguimos,” “trabajando”), (“Hay,” “bendiciones”), (“Continuamos,” “afirmaciones”), (“MEREZCO,” “…”), (“salud,” “tod”), (“todxs,” “ME”), (“bendiciones,” “todxs”), (“días,” “todxs”), (“todxs,” “Continuamos”), (“amig,” “@”), (“s,” “seguimos”), (“-Jueves,” “20”), (“-Sábados,” “22…”), (“-Viernes,” “22”), (“03hs,” “-Sábados”), (“12hs,” “-Viernes”), (“15/03,” “Horarios”), (“20,” “12hs”), (“22,” “03hs”), (“Enamoradxs,” “puesto”), (“Horarios,” “-Jueves”), (“Reclamá,” “descuento”), (“aportar,” “granito”), (“descuento,” “15/03”), (“granito,” “arena”), (“Esta,” “noche”), (“Se,” “acerca”), (“puesto,” “queremos”), (“queremos,” “aportar”), (“acerca,” “Día”), (“Feliz,” “día”), (“mujeres,” “ 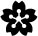 Soy”), (“33,” “Aprovechamos”), (“Aprovechamos,” “felicitarlxs”), (“Estuvo,” “genial”), (“Jinx,” “color”), (“**NicoOviedomusic**,” “primera”), (“Personas,” “Trans”), (“Webconf,” “acompañando”), (“Wil_Sound,” “Estan”), (“acá,” “video”), (“azul,” “ 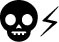 ”), (“beerjscba,” “edición”), (“calentando,” “pista”), (“color,” “azul”), (“conexionhiphop,” “Estuvo”), (“evento,” “ 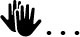 …”), (“excelente,” “evento”), (“felicitarlxs,” “excelente”), (“primera,” “Living”), (“todxs 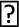 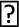 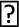 ,” “Jockey”), (“lxs,” “Enamoradxs”), (“¡Buen,” “día”), (“s,” “l”), (“Hola,” “amig”), (“Living,” “año”), (“Si,” “problemitas”), (“comienzo,” “semana”), (“noche,” “calentando”), (“video,” “show”), (“ 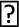 ,” “Si”), (**“Jockey,” “Club”**), (“Viernes,” “5/10/18”), (“Buen,” “comienzo”), (“ 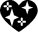 18M,” “Día”), (“día,” “Se”), **(“Nueva,” “Córdoba”)**, (“arena,” “de…”), (“año,” “junto”), (“Artistas,” “Y”), (“Y,” “acá”), (“Día,” “lxs”), (“Dando,” “bendiciones”), (“bendiciones,” “todxs 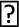 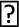 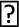 ”), (“¡Feliz,” “día”), (“@,” “Reclamá”), (“@,” “s.”)]
**GBALP**	
[(“@,” “s”), (**“Buenos,” “Aires”**), (“tod,” “@”), (“día,” “espartan”), (“buen,” “día”), (“amig,” “@”), (“espartan,” “@”), (**“Ciudad,” “Autónoma”**), (“l,” “@”), (“ 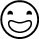 ,” “buen”), (**“Autónoma,” “Buenos”**), (“@,” “s 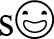 ”), (“todxs,” “lxs”), (“lxs,” “compañerxs”), (“Gracias,” “todxs”), (“Feliz,” “día”), (“4,” “ 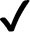 ”), (“#,” “#”), (“ 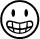 ,” “buen”), (“ 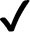 ,” “ 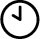 10”), (“ 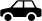 50,” “n°”), (“CUOTAS,” “SIN”), (“n°,” “436 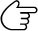 ”), (“#,” “indumentaria”), (“indumentaria,” “#”), (“3,” “4”), (“ 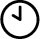 10,” “19 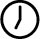 ”), (“Buenas,” “noches”), (**“Plaza,” “Mayo”**), (**“Florencio,” “Varela”**), (“6,” “CUOTAS”), (“lxs,” “trabajadorxs”), (**“La,” “Plata”**), (“436 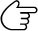 ,” “3”), (“SIN,” “INTERÉS”), (“AHORA,” “3”), (“Distrito,” “Federal”), (“@,” “#”), (“lxs,” “vecinxs”), (“#,” “ropacononda”), (“ropacononda,” “#”), (“lxs,” “chicxs”), (“Nos,” “vemos”), (“ell,” “@”), (“#,” “hombres”), (“hombres,” “#”), (“Aires,” “Argentina”), (“Se,” “viene”), (“lxs,” “pibxs”), (“Y,” “6”), (“s,” “l”), (“@,” “s.”), (**“Ramos,” “Mej**í**a”**), (“#,” “ropa**laplata**”), (“ropa**laplata**,” “#”), **(“Bs,” “As”)**, (“compañer,” “@”), (“día,” “maravilloso”), (“Locas,” “Ni”), (“Ni,” “Locas”), (“Ni,” “Santas”), (“3,” “Y”), **(“San,” “Miguel”)**, (“maravilloso,” “ 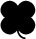 ”), (“Buen,” “día”), (“MUEVELO,” “3D”), (“glewsolidaria,” “Diferentes”), (“ 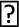 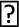 ,” “Pedí”), (“#,” “**laplata**”), (“alumn,” “@”), (“Diferentes,” “Instituciones”), (“Glew,” “unidos”), (“fin,” “semana”), **(“Capital,” “Federal”)**, (“Hola,” “amig”), (“s 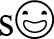 ,” “ 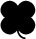 ”), (“#,” “@”), **(“Varela,” “Buenos”)**, (“unidos,” “ayudar”), (“Acaba,” “publicar”), (“romantico,” “brillo”), (“#,” “ropalaplata…”), (“Muchas,” “gracias”), (“Gracias,” “tod”), (“brillo,” “propio”), (“cruzrojaargalmirantebrown,” “acompañarnos”), (“original,” “¡Durante”), (“quedateencasa,” “ALUMNA”), (“regala,” “original”), (“necesita,” “una…”), (“Desafiocultural,” “tema”), (“tema,” “RESISTIRE”), (“@,” “s 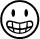 ”), (“vecin,” “@”), (“Instituciones,” “vecin”), (“brindar,” “charlas…”), (“jornadasolidaria,” “brindar”), **(“Puerto,” “Madero”)**, (“#,” “esparta”)]	

**Table 3:** Frequency list of single tokens.

**BA**	**Córdoba**
[(“@,” 582), (“s,” 438), (“todxs,” 303), (“lxs,” 302), (“Gracias,” 161), (“tod,” 148), (“amigues,” 139), (“día,” 126), (“Buenos,” 106), (“amig,” 104), (“amigxs,” 95), (“Aires,” 92), (“Ciudad,” 79), (“La,” 79), (“l,” 77), (“Feliz,” 73), (“Autónoma,” 69), (“Hoy,” 59), (“compañerxs,” 58), (“…,” 57), (“Y,” 54), (“año,” 51), (“semana,” 47), (“En,” 45), (”““,” 44), (“gracias,” 42), (“chiques,” 42), (“junto,” 39), (“Con,” 39), (“siempre,” 39), (“ellxs,” 35), (“noche,” 34), (“viernes,” 34), (“mejor,” 33), (“Buen,” 33), (**“Plaza,”** 33), (“esperamos,” 33), (“Argentina,” 33), (“Que,” 32), (“amor,” 31), (“trabajadorxs,” 31), (“hoy,” 30), (“de…,” 30), (“ser,” 30), (“sábado,” 29), (“si,” 29), (“vida,” 28), (“Este,” 28), (“..,” 27), (“show,” 27), (“DE,” 26), (“amigos,” 26), (“cada,” 26), (“Les,” 26), (“No,” 26), (“en…,” 25), (““,” 25), (“dia,” 25), (“años,” 25), (“Una,” 25), (“vez,” 25), (“Si,” 24), (“días,” 24), (“nuevo,” 24), (“De,” 24), (“Los,” 24), (“Ayer,” 24), (“**Mayo**,” 22), (“gran,” 22), (“Para,” 22), (“Un,” 22), (“Día,” 22), (“Noche,” 22), (“e…”, 22), (“Federal,” 22), (“lindo,” 21), (“domingo,” 21), (“x,” 21), (“a…,” 20), (“Se,” 20), (“anoche,” 20), (“ 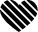 ,” 20), (“nuestrxs,” 19), (“y…,” 19), (“ 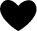 ,” 19), (“**Palermo**,” 19), (“Por,” 19), (“feliz,” 19), (“amigx,” 19), (“jueves,” 18), (“Hola,” 18), (“nosotrxs,” 18), (“hermosa,” 18), (“fin,” 18), (“Teatro,” 18), (“2019,” 18), (“viene,” 17), (“.…,” 17), (“ver,” 17)]	[(“@,” 53), (“s,” 53), (“todxs,” 32), (“tod,” 28), (“l,” 18), (“día,” 18), (“lxs,” 17), (“…,” 16), (“bendiciones,” 13), (“Hay,” 12), (“En,” 12), (“seguimos,” 12), (“Buenos,” 11), (“serie,” 11), (“días,” 10), (“amig,” 10), (“Córdoba,” 10), (“LO,” 10), (“tratamientos,” 10), (“dejó,” 10), (“querida,” 10), (“**Louise**,” 10), (“L,” 10), (“amigxs,” 9), (“afirmaciones,” 9), (“positivas,” 9), (“mujeres,” 9), (“Seguimos,” 9), (“ME,” 9), (“MEREZCO,” 9), (“Plan,” 9), (“**X5,”** 9), (“prioridad,” 9), (“salud,” 9), (“trabajando,” 9), (“manera,” 9), (“ONLINE,” 9), (“atender,” 9), (“todas…,” 9), (“Continuamos,” 8), (“amigues,” 7), (“““,” 7), (“Y,” 7), (“Hoy,” 5), (“Buen,” 5), (“hoy,” 5), (“Día,” 5), (“..,” 5), (“Feliz,” 5), (“Se,” 4), (“espero,” 4), (“compañerxs,” 4), (“Club,” 4), (“hermoso,” 4), (“Viernes,” 4), (“Que,” 4), (“amor,” 4), (“LA,” 4), (“Esta,” 4), (“queremos,” 4), (“gracias,” 4), (“La,” 4), (“ 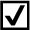 ,” 4), (“Hola,” 4), (“pasó,” 3), (“lleno,” 3), (“Ellxs,” 3), (“Gracias,” 3), (“el…,” 3), (“semana,” 3), (“quiero,” 3), (“Este,” 3), (“Mañana,” 3), (“junto,” 3), (“show,” 3), (“s…,” 3), (“O,” 3), (“noche,” 3), (“año,” 3), (“¡Buen,” 3), (“acerca,” 3), (“Enamoradxs,” 3), (“puesto,” 3), (“aportar,” 3), (“granito,” 3), (“arena,” 3), (“de…,” 3), (“De,” 3), (“mejor,” 3), (“formación,” 3), (“s.,” 3), (“ 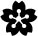 Soy,” 3), (“Si,” 3), **(“Córdoba,” 3), (“Nueva,”** 3), (“sábado,” 3), (“ 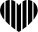 ,” 3), (“Reclamá,” 3), (“descuento,” 3)]
**GBALP**	
[(“@,” 843), (“s,” 841), (“lxs,” 541), (“todxs,” 494), (“día,” 456), (“tod,” 270), (“Gracias,” 262), (“amig,” 201), (“**Buenos**,” 189), (“amigues,” 184), (“buen,” 173), (“espartan,” 167), (“**Aires**,” 165), (“La,” 147), (“l,” 141), (“amigxs,” 138), (“Hoy,” 138), (“Feliz,” 134), (“compañerxs,” 124), (“ 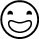 ,” 123), (“Y,” 107), (“…,” 98), (“**Ciudad**,” 84), (“año,” 84), (“gracias,” 81), (“En,” 81), (“semana,” 79), (“Buen,” 77), (“chiques,” 76), (“junto,” 75), (“**Autónoma**,” 69), (“,” 68), (“siempre,” 67), (“Con,” 67), (“viernes,” 65), (“s 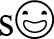 ,” 65), (“Los,” 64), (“Que,” 63), (“hoy,” 62), (“amor,” 57), (“..,” 56), (“3,” 55), (“si,” 55), (“vecinxs,” 55), (“sábado,” 54), (“esperamos,” 54), (“**Argentina,”** 54), (“mejor,” 52), (“noche,” 52), (“de…,” 50), (“No,” 50), (“Este,” 49), (“años,” 49), (“San,” 48), (“indumentaria,” 48), (“vida,” 47), (“feliz,” 47), (“ser,” 46), (“cada,” 46), (“ 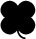 ,” 46), (“DE,” 45), (“dia,” 45), (“ellxs,” 45), (“días,” 44), (“en…,” 44), (“Les,” 44), (“Un,” 44), (“4,” 44), (“Para,” 42), (“De,” 42), (“Hola,” 41), (“trabajadorxs,” 40), (“Lxs,” 40), (“gran,” 38), (“domingo,” 38), (“**Plaza**,” 38), (“nosotrxs,” 38), (“vez,” 38), (“Si,” 36), (“Se,” 36), (“Por,” 36), (“ver,” 35), (“amigos,” 35), (“a…,” 35), (“nuevo,” 35), (“Una,” 35), (“tan,” 35), (“show,” 34), (“e…,” 34), (“x,” 33), (“ropacononda,” 33), (“LA,” 32), (“jueves,” 32), (“amigx,” 32), (“Día,” 32), (“gente,” 32), (“q,” 32), (“.…,” 32), (“ 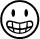 ,” 32)]	

The lists of most frequent words and collocations in Tables 2, 3 clearly show a preference for plural forms of gender-neutral tokens. Most frequent nouns that appear with *-@/-x/-e* are listed in order of their frequency: *amigxs, compañerxs, trabajadorxs*, “friends_[neut]_, companions_[neut]_, workers_[neut]_.” GBALP contains other lexical nouns: *espartan@s, vecinxs, chic@s, pibxs, alumn@s, niñxs* “people_[neut]_ associated with the clothing brand *Esparta*, neighbors_[neut]_, girls_[neut]_, guys/girls_[neut]_, students_[neut]_, kids_[neut]_.” Most nouns express familiarity such as “friends, companions, neighbors.” The familiarity relationship is also marked by informal register, such as “guys.”

The most frequent speech act expresses the addressing of the aforementioned groups of people in order to thank them and to wish them a good day. This is shown by the most frequent collocations and tokens: “Thank you/thanks to,” “Happy day/Saturday” in Tables 2, 3 and illustrated by examples (1–5). Another of the most frequent speech acts is informing the addressee about a future or past event (e.g., the release of a new album, as in 2, or a course for fashion entrepreneurs in 5) and inviting them to this event. This interpretation follows from the most frequent lexical words such as: “follow (us)!,” “we will see each other,” “we wait for you,” the use of event nouns and temporal expressions: “tonight, event, music, show.”

(1) Gracias a todxs mis amigxs que me acompañaron en la tercera #función….
  “Thanks to all my friends who accompanied me to the third #function….”
(2) Queridos amigxs! Seguimos presentando el nuevo EP: “Soltando mi Cruz.”
  “Dear friends! We continue to present the new EP: ‘Releasing my cross'.”
(3) Buen sábado para todxs!!! #workinprogress en Ganesha Art Tattoo Studio.
  “Good Saturday to everyone!!! #workinprogress at Ganesha Art Tattoo Studio.”
(4) Gracias amigues! Nos vemos prontito! en Teatro Gastón.
  “Thank, you friends! We'll see each other very soon! In the Gastón Theater.”
(5) '¡Atenti a todxs! !
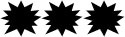
. 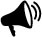 Se acerca un nuevo curso para los ¡EMPRENDEDORES DE MODA! 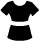 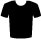 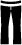 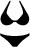 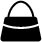 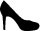. Y, además, en PRE-VEN….
  “Attention everyone! We are approaching the deadline of the course for fashion entrepreneurs….”

Given the intention of the writers to inform people about an event and invite them to it, the use of plural nouns in the form of address is a logical move: plural implies more people than singular.

The most frequent speech acts are as follows: (1) addressing people with a familiarity relationship, (2) wishing them a good day, (3) informing them about an event or a product/service, and (4) inviting them to an event or store.

In the next step, I will look into tokens in the frequency lists representing names. The names are listed here in the order of their frequency:

(6) BA: 1.Plaza de Mayo, 2. San Telmo, 3. Puerto Madero, 4. Teatro Río (Colorado), 5. Junta Interno, 6. Ganesha Art (Tattoo Studio), 7. Junta. 8. Facultad Direcha, 9. Caballito, 10. Club 69, 11. Botica Ángel, 12. Medrano, 1647, 13. Norita Cortiñas, 14. Partido Piquetero, 15. Tango Butoh, 16. Palermo, Soho.
(7) Córdoba: 1. Louise, 2. Oviedomusic. 3. Jockey Club, 4. Nueva Córdoba.
(8) GBALP: 1. Buenos Aires. 2. Plaza de Mayo. 3. Florencio Varela, 4. La Plata and 5. Ramos Mejia, 6. San Miguel, 7. Puerto Madero.

The names of locations mentioned in BA and Córdoba have the common characteristics of being in the city center. Plaza de Mayo is a square where people gather to demonstrate. It is close to the presidential palace in Buenos Aires. In fact, all tweets containing a reference to Plaza de Mayo are related to demonstrations:

(9) Marchando a Plaza de Mayo en unidad con lxs trabajadorxs 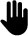 #BastaDeAjuste #NOalFMI #FrenemosElAjuste.
  “Marching to the Plaza de Mayo in unity with the workers 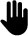 #Enough Adjustment #No to the IMF #Stop the Adjustment.”
(10) Marcho como aliada. Marcho con orgullo. Marcho por lxs que no pueden. @ Plaza de Mayo.
  “I march as an ally. I march with pride. I march for those who cannot. @ Plaza de Mayo.”

Other names of locations associated with demonstrations or political statements are *Junta Interno*, as shown in the list in (6). This name is mentioned as a starting point for a political demonstration (see “From Junta” in Table 2 left). Other lexical elements associated with demonstrations and political activism in Tables 2, 3 are *trabajadores* “workers,” *trabaj-* “work,” INCA (name of an ethnic minority), “solidarity,” LNP (a political party), “the Piquetero party,” “Faculty of Law.” An example is provided as follows:

(11) Día Internacional de la Mujer. Marchando por mis derechos y los de todxs los demás.
  “International Women's Day. Marching for my rights and everyone else's.”

The most frequent tweets from Córdoba are associated with female activism. These tweets contain messages about mental strength for women published by a female writer Louise L Hay:

(12) Hoy comenzamos con una serie de tratamientos que nos dejó la querida Louise L Hay, bendiciones a todxs!!!A seguir….
  “Today we will start with a series of treatments that were provided for us by the beloved Louise L Hay, blessings to everyone[neut]!!! To continue….”
(13) Buenos días para todxs!! Comenzamos con una serie de afirmaciones positivas para las mujeres. 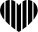
LAS AFIRMACIONES SON.
  “Good morning to all! We begin with a series of positive affirmations for women. 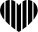
THE AFFIRMATIONS ARE.”

Recall that activism plays an important role in the literature on GNL (Section Introduction).

However, activism is not the only topic of the tweets with GNL. The list of names in Tables 2, 3 also contain names of businesses, such as *Ganesha Art Tattoo, Hairsaloon*, and *X5* (car engineering company), as well as entertainment venues such as *Theater, Tango Butoh, Club 69, oviedomusic* (sound producer), *Jockey Club* (sports club), and neighborhoods such as Caballito and Palermo famous for their bars, cafes, and music events. A common characteristic of companies in BA and GBALP is that they sell products or services related to personal appearance: clothing, hairstyling, and tattoos. In Córdoba, companies that use GNL are related to technology.

The collocations representing GBALP also mention smaller cities than BA such as Florencio Varela, La Plata, Ramos Mejía, and San Miguel. Most tweets from La Plata are associated with the brand *Esparta*, hence the use of *espartan@s* in Tables 2, 3, which happens to be one of the most frequent tokens. This indicates that the most frequent tokens in GBALP are coming from La Plata and more precisely from the company *Esparta*:

(14) “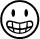 buen día espartan@s
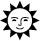
hermoso Lunesss 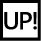 Entró la bomber térmica 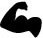

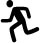
\u200d♂
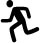
\u200d♀dale que vuelan
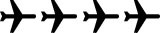
 Pedí un Glovo….
  “Hello, Esparta fans_[neut]_ Have a nice Monday. A thermal bomber jacket just came in. Hurry up! Order with the Glovo app.”
(15) “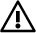 Atención Reyes magos espartan@s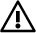 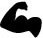 Mañana sábado los esperamos de 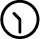
10:30 a 19 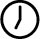 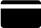 AHORA 3 Y 6 CUOTAS SIN INTERÉ….
  “Attention, Esparta Wise Men_[neut]_! Tomorrow on Saturday we wait for you from 10.30 to 19.00 Now pay in 3 or 6 installments interest-free.”

The observation that most frequent tweets with GNL are coming from La Plata and not bigger cities such as BA and Córdoba is unexpected, given that La Plata is four times smaller than BA and two times smaller than Córdoba with respect to the number of inhabitants. This shows that the frequency distribution of tweets with GNL does not reflect the size of a city measured by the number of inhabitants.

To summarize, the textual analysis of the frequency lists of single tokens and collocations has shown that most tweets with gender-neutral expressions are used for greeting people with whom the writer of the tweet is familiar, to wish them a good day and/or to provide them with information about events, products, or services and invite them to events or stores where the products/services are sold. The information about the events can further be subdivided into different groups: commercial, political (demonstration, activism), cultural (theater, music events), and social (neighborhood events). The types of events vary from city to city. Commercial events are more frequent in La Plata, whereas tweets related to social intent and political engagement are more frequent in BA and Córdoba.

### Temporal Information

Temporal information can be used to identify particular events that play an important role in usage of GNL.

Recall from the background concerning political events in Argentina in Section Introduction, that according to the Washington Post, GNL gained official acceptance in 2019 after the social debate triggered by criticism of a young journalist who used GNL in an official interview in 2018. The year 2019 was also important for the elections as it is the year when Alberto Fernández, a supporter of GNL usage, became president.

This background is reflected in the temporal changes in Table 4.

**Table 4:** Frequency of ± neutral gender expressions per year.

**Time**	**GBALP**	**BA**	**Córdoba**
2018			
**+neutral** Gendered	**758 = 0.35%** 213,028 = 99.65%	**510 = 0.39%** 131,609 = 99.61%	**33 = 0.31%** 10,523 = 99.69%
2019			
**+neutral** Gendered	**1189 = 0.58%** 202,509 = 99.42%	**615 = 0.56%** 109,390 = 99.44%	**70 = 0.53%** 13,038 = 99.47%
2020			
**+neutral** Gendered	**770 = 0.56%** 135,758 = 99.44%	**335 = 0.48%** 69,871 = 99.52%	**32 = 0.32%** 9,777 = 99.68%

There is a clear rise in frequency from 2018 to 2019 of GNL usage in all areas investigated and a slight fall in 2020. The rise of GNL usage could have been caused by the official recognition of gender-neutral expressions in 2019. We see a difference in frequency distribution between GBALP and the two big cities BA and Córdoba. The fall of gender-neutral usage is greater in BA and Córdoba than in GBALP. We also see a fall in the absolute numbers of geolocated tweets representing the use of (overt) gendered expressions. This fall could be due to Twitter's decision to restrict geolocation information noted in Section Data and Methods.

We now zoom in and look at the distribution of gender-neutral usage per month.

**Figure 1:**
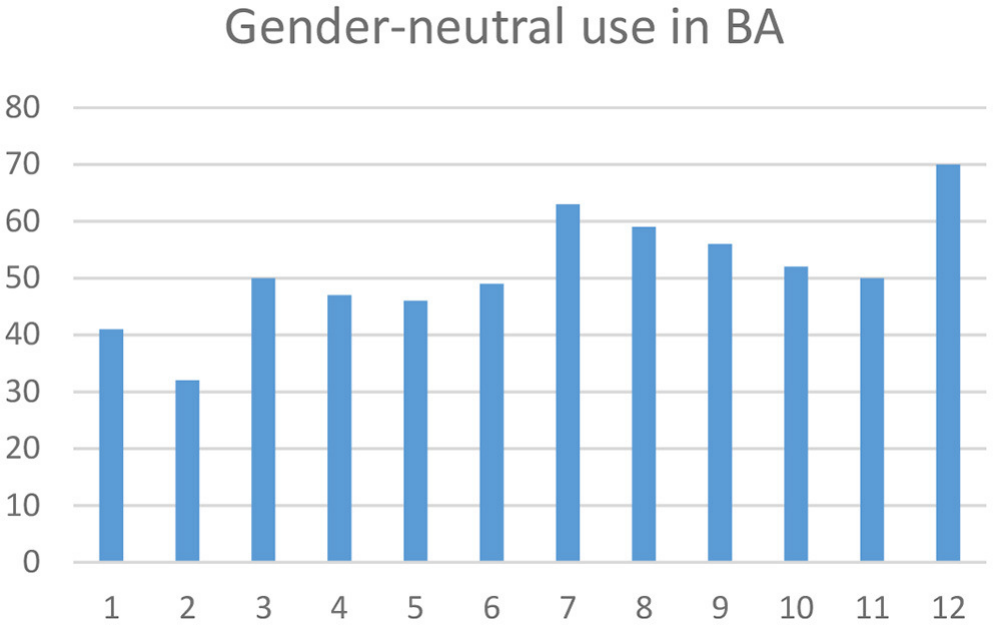
Timeline of gender-neutral usage: x axis = months, and y axis = frequency of tweets.

Figure 1 shows the distribution of gender-neutral tweets per month in 2019 in BA. There are some differences in frequency. From January on, I see some rises in March, July, and December.

I analyzed the tweets in these months and have identified the most frequent speech acts in these tweets, namely greetings and wishes for Christmas/New Year's Eve in December, for *D*í*a de la Independenci*a “Independence Day” and *D*í*a del Amigo* Friends Day in July, and for *D*í*a Internacional de la Mujer* “Women's Day” in March. Here are some examples:

(16) Feliz día del amig@ a tod@s… los que están lejos o cerca, a los que veo seguido, a los que no veo nunca… “Happy Friends Day[neut] to all[neut] who are far away or close, to those who I always see and never see….”
(17) Feliz Navidad para ustedes que me siguen, para mis amigxs, compañerxs, colegas, a mi familia…
  “Happy Christmas to you that follow me, to my friends, companions, colleagues, to my family….”

The content analysis fits with the rise of tweets in particular months and preceding specific events in these months in BA.

**Figure 2:**
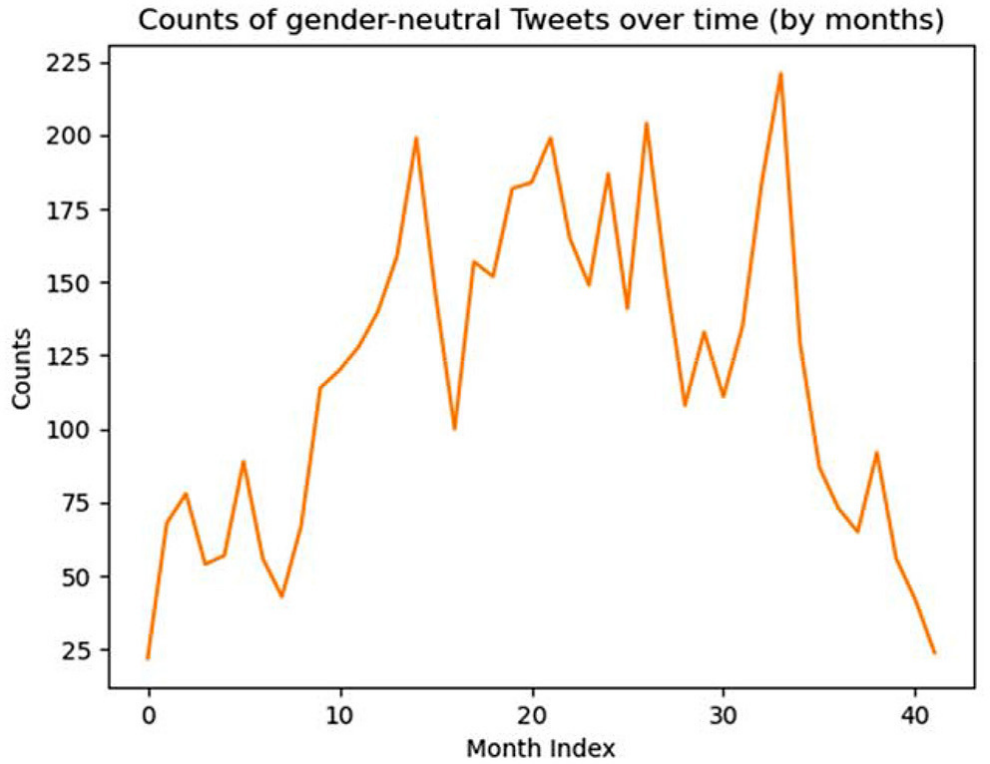
Frequency of gender-neutral expressions by month in GBALP.

**Figure 3:**
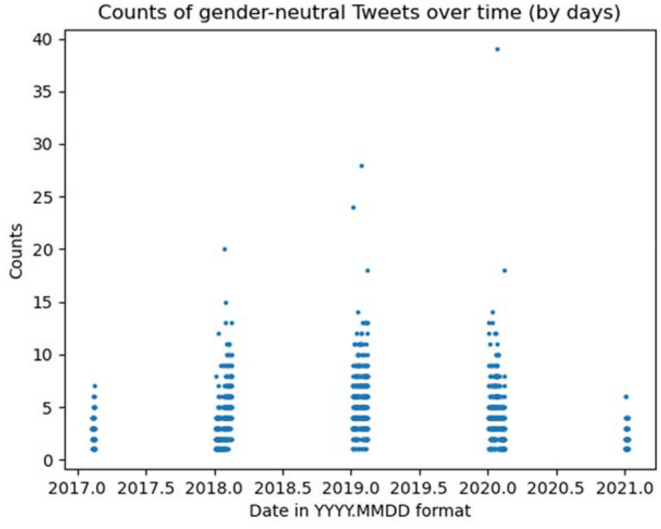
Frequency of gender-neutral usage by day in GBALP.

If we now look at the distribution of tweets by month in GBALP in Figure 2, we see a particular spike in the 33rd month, which corresponds to July 2020. If we zoom in on the distribution per day, we see a point representing a day in 2020 that has the highest frequency number, with 37 tweets (see Figure 3). Except for this outlier, the distribution of tweets in 2020 is very similar to that in 2019. This day, July 20, happens to be the Friends Day.

Our temporal analysis thus confirms that the most important function of tweets with gender-neutral usage is to greet people and wish them all the best for important national and international days.

## Discussion and Outlook

Our results confirm the hypothesis from the literature that GNL usage is associated with the urban environment and upper- and middle-class areas (see Moure, 2018; Tosi, 2021). However, our data do not confirm that GNL is only used in the realms of activism and certain public administration sectors (Moure, 2018; Tosi, 2021), at least not in the geolocated tweets. Our results show that the commercial sector is an important domain of gender-neutral usage. Cultural and social contexts are other important contexts of gender-neutral usage. Tweeters address people of all genders to invite them to neighborhood and cultural events without any explicit political message. Political activism is not the only social context of gender-neutral usage in social media. Our temporal analysis confirms this analysis showing that the frequency of gender-neutral usage is the highest in months and days that correlate with important national and international days and festivities. Twitter users greet and congratulate friends and other groups with close social relationship for these important days, such as Christmas and Friends Day.

Our goal for the future research is to continue to observe gender-neutral usage in the long term, as this phenomenon is very recent and we might observe a social and a linguistic change in the future. A social change might include more users of different social groups than presently observed. A linguistic change might include effects of grammaticalization, such as when gender-neutral markers lose their function of inclusive gender and start being used in a broader linguistic context (e.g., with a wider range of nouns). An interesting observation that needs further investigation in the future is that gender-neutral markers agree with other linguistic elements in gender-neutrality to various degrees:

(18)   tod@s los amigos. “all friends”     No agreement.
(19)   tod@s l@s fotograf@s “all fotographers”     Full agreement.
(20)   tod@s los enamord@s “all lovers”     Partial agreement.

I interpret the variation in agreement as a mirror of uncertainty in how to use gender-neutral markers. This conclusion needs further observation and testing in the future research. Shroy (n.d) has also observed a high variation in agreement in GNL in French. A systematic comparison with other languages that use gender-neutral markers such as French (Shroy, n.d) and other Spanish speaking countries would definitely provide more information about this phenomenon as well as the variation in agreement. In the future, I will explore computational methods for filtering out all potential linguistic elements that have a person feature in order to filter out all elements that do have or do not have a gender-neutral marker. I will study computational methods that differentiate between animate and inanimate nouns (Orǎsan and Evans, 2007; Heng and Dekang, 2009).

The present study does not include doubled forms (e.g., *los chicos y las chicas* “the boys and the girls”) as a form of GNL, which is the second most popular form in oral speech according to Slemp (2020), nor does the present study include the form -e with the exception of few tokens (*amigues* and *chiques*). It is highly interesting how different the language context of GNL is in oral speech in comparison to written speech, which is the focus in the present study.

We also aim at analyzing social context information on the basis of geolocation information. Geolocation information will provide us with answers as to where exactly tweets with gender-neutral usage come from and whether these locations match our description of the social context of gender-neutral usage based on textual analysis.

## Data Availability Statement

The original contributions presented in the study are included in the article/Supplementary Material, further inquiries can be directed to the corresponding author.

## Author Contributions

The author confirms being the sole contributor of this work and has approved it for publication.

## Funding

The costs of the article will be paid by our library supporting open access publication fees.

## Conflict of Interest

The author declares that the research was conducted in the absence of any commercial or financial relationships that could be construed as a potential conflict of interest.

## Publisher's Note

All claims expressed in this article are solely those of the authors and do not necessarily represent those of their affiliated organizations, or those of the publisher, the editors and the reviewers. Any product that may be evaluated in this article, or claim that may be made by its manufacturer, is not guaranteed or endorsed by the publisher.

## Footnotes

^1^Kellert, O., and Matlis, N. H. (under review). Geolocation of multiple sociolinguistic markers in Buenos Aires. PLOS One.
