# Supplementary material for: Gender Neutral Language in (Greater) Buenos Aires, (Greater) La Plata, and Córdoba: An Analysis of Social Context Information Using Textual and Temporal Features
Source: Front Sociol. 2022 Mar 17;7:805716. doi: 10.3389/fsoc.2022.805716 (PMC8969771; doi:10.3389/fsoc.2022.805716)
Supplement: Supplementary file 1 [file Table_1.DOCX]

English translations of Table 2 and Table 3

Buenos Aires

[('@', 's'), ('Buenos', 'Aires'), ('all', '@'), ('Ciudad', 'Autónoma'), ('friend', '@'), ('Autónoma', 'Buenos'), ('l', '@'), ('all', 'they'), ('Thanks', 'all'), ('Happy, 'day'), ('Plaza', 'Mayo'), ('Distrito', 'Federal'), ('the', 'workers'), ('you', 'we will see'), ('the', companions'), ('Bs', 'As'), ('Capital', 'Federal'), ('many', 'thanks'), ('San', 'Telmo'), ('Puerto', 'Madero'), ('end', 'week'), ('Río', 'Colorado'), ('s', 'l'), ('LNP', 'San'), ('the', 'guys'), ('ell', '@'), ('Theater', 'Rio'), ('Art', 'Tattoo'), ('Ganesha', 'Art'), ('FASHION', '👚'), ('follow', 'automatically'), ('Tattoo', 'Studio'), ('automatically', 'participate.... '), ('one-man', 'payase'), ('👖', '👙'), ('👙', '👜'), ('👜', '👜', '👠'), ('💥💥💥', '📣Se'), ('Colorado', 'theater/school'), ('Board', 'Intern'), ('ME', 'LIKE'), ('also', 'PRE-VEN.... '), ('pongas', 'ME'), ('I kepp', 'singing'), ('🔽🔽🔽🔽🔽🔽🔽', 'follow us'), ('curso', '¡entrepreneurs'), ('on', 'line'), ('greet', 'thank'), ('✔', 'Soy') ('👕', '👖'), ('👚', '👕'), ('the', 'girls'), ('Telmo', 'Ciudad'), ('we invite', 'put'), ('From', 'Junta'), ('like', 'us'), ('us', 'follow'), ('I want', 'greet'), ('📣Se-refl', 'coming soon'), ('we', '@'), ('L', '@'), ('Federal', 'Argentina'), ('69', 'peace'), ('wine', 'only'), ('Faculty', 'Law'), ('Good', 'day'), ('#', '@'), ('first', 'time'), (workers', 'INCAA'), ('Law', 'UBA'), ('Me', 'follow'), ('Caballito', 'Buenos'), ('Club', '69'), ('just', 'posted'), ('Botica', 'angel'), (Make up, 'Professional'), ('Medrano', '1647'), ('NIKE', 'ZOOM'), ('Norita', 'Cortiñas'), ('Partido', 'Piquetero'), ('TANGO', 'BUTOH'), ('Trayecto', Make up), ('ZOOM', 'HYPERATACK'), ('I would say', '🥰🌠💛🌊'), ('age', 'levels'), ('elisabetsauro', 'pinallieugenioangel'), ('tribute', 'standard-bearer'), ('import', 'ages'), ('professionals‼', 'find them'), ('psychomotor', 'fine'), ('Angel', '😇🎭'), ('companiont', '@'), ('"', 'I want'), ('good', 'wave'), ('Palermo', 'Soho'), ('Very', 'happy'), ('Etiquette', 'friend'), ('Find them', 'store')]

Córdoba

[('@', 's'), ('all', '@'), ('Louise', 'L'), ('let', 'dear'), ('dear', 'Louise'), ('treatments', 'left'), ('Good', 'days'), ('series', 'treatments'), ('l', '@'), ('ONLINE', 'attend'), ('Plan', 'X5'), ('X5', 'priority'), ('affirmations', 'positive'), ('attend', 'all.... '), ('way', 'ONLINE'), ('positive', 'women'), ('priority', 'health'), ('working', 'way'), ('it', 'There is'), ('IT', 'I DESERVE'), ('ME', 'IT'), ('We continue', 'series'), ('In', 'Plan'), ('we continue', 'working'), ('There', 'blessings'), ('We continue', 'affirmations'), ('I DESERVE', '.... '), ('health', 'all'), ('all', 'ME'), ('blessings', 'all'), ('days', 'all'), ('all', 'We continue'), ('friend', '@'), ('s', 'we continue'), ('-Thursday', '20'), ('-Saturday', '22.... '), ('-Friday', '22'), ('03hs', '-Saturdays'), ('12hs', '-Friday'), ('15/03', 'Schedules'), ('20', '12hs'), ('22', '03hs'), (‘lovers’, 'post'), ('Schedules', '-Thursday'), ('Ask', 'descuento'), ('contribute', 'granito'), ('descuento', '15/03'), ('granito', 'sand'), ('This', 'night'), ('Se-refl.', 'coming close'), ('position', 'we want'), ('we want', 'bring'), ('close', 'Day'), ('Feliz', 'día'), ('women', '🌸Soy'), ('33', 'take advantage of the moment'), ('take advantage of the moment', 'congratulate'), ('Was', 'super'), ('Jinx', 'colour'), ('NicoOviedomusic', 'first'), ('People', 'Trans'), ('Webconf', 'accompanying'), ('Wil_Sound', 'are'), ('here', 'video'), ('blue', '💀⚡'), ('beerjscba', 'edition'), ('warming up', 'track'), ('color', 'blue'), ('conexionhiphop', 'was'), ('event', '👏.... '), ('excellent', 'event'), ('congratulatethem', 'excellent'), ('primery', 'Living'), ('todxs🙏🏻😂', 'Jockey'), ('the', 'lovers'), ('¡Good', 'day'), ('s', 'l'), ('Hello', 'friend'), ('Living', 'year'), ('Si', 'problemitas'), ('start', 'week'), ('night', 'warming up'), ('video', 'show'), ('🖤', 'Yes'), ('Jockey', 'Club'), ('Friday', '5/10/18'), ('Good', 'start'), ('💖18M', 'Day'), ('day', 'Se'), ('New', 'Córdoba'), ('arena', 'de.... '), ('year', 'together'), ('Artists', 'And'), ('And', 'here'), ('Day', 'lxs'), ('Giving', 'blessings'), ('blessings', 'all🙏🏻😂'), ('Happy', 'day'), ('@', 'Reclaim'), ('@', 's. ')]
